# Cadmium-induced apoptosis of Leydig cells is mediated by excessive mitochondrial fission and inhibition of mitophagy

**DOI:** 10.1038/s41419-022-05364-w

**Published:** 2022-11-05

**Authors:** Lingna Yi, Xue-Jun Shang, Linglu Lv, Yixiang Wang, Jingjing Zhang, Chao Quan, Yuqin Shi, Yunhao Liu, Ling Zhang

**Affiliations:** 1 School of Public Health, Hubei Province Key Laboratory of Occupational Hazard Identification and Control, Wuhan University of Science and Technology, Wuhan, 430065 China; 2 Department of Urology, Jinling Hospital Affiliated to Nanjing University School of Medicine, Nanjing, 210002 China

**Keywords:** Apoptosis, Infertility

## Abstract

Cadmium is one of the environmental and occupational pollutants and its potential adverse effects on human health have given rise to substantial concern. Cadmium causes damage to the male reproductive system via induction of germ-cell apoptosis; however, the underlying mechanism of cadmium-induced reproductive toxicity in Leydig cells remains unclear. In this study, twenty mice were divided randomly into four groups and exposed to CdCl_2_ at concentrations of 0, 0.5, 1.0 and 2.0 mg/kg/day for four consecutive weeks. Testicular injury, abnormal spermatogenesis and apoptosis of Leydig cells were observed in mice. In order to investigate the mechanism of cadmium-induced apoptosis of Leydig cells, a model of mouse Leydig cell line (i.e. TM3 cells) was subjected to treatment with various concentrations of CdCl_2_. It was found that mitochondrial function was disrupted by cadmium, which also caused a significant elevation in levels of mitochondrial superoxide and cellular ROS. Furthermore, while cadmium increased the expression of mitochondrial fission proteins (DRP1 and FIS1), it reduced the expression of mitochondrial fusion proteins (OPA1 and MFN1). This led to excessive mitochondrial fission, the release of cytochrome c and apoptosis. Conversely, cadmium-induced accumulation of mitochondrial superoxide was decreased by the inhibition of mitochondrial fission through the use of Mdivi-1 (an inhibitor of DRP1). Mdivi-1 also partially prevented the release of cytochrome c from mitochondria to cytosol and attenuated cell apoptosis. Finally, given the accumulation of LC3II and SQSTM1/p62 and the obstruction of Parkin recruitment into damaged mitochondria in TM3 cells, the autophagosome-lysosome fusion was probably inhibited by cadmium. Overall, these findings suggest that cadmium induces apoptosis of mouse Leydig cells via the induction of excessive mitochondrial fission and inhibition of mitophagy.

## Introduction

In recent decades, sperm quality and levels of characteristic hormones such as testosterone in males have continued to decline [1]. The decrease in quality of male semen may be attributed to several causes, notably changes in lifestyle, environmental pollution, genetic factors, and even issues around food safety [2, 3]. The health and environmental implications of cadmium exposure have, nonetheless, received increasing attention, particularly given the frequent deployment of cadmium in applications such as battery production, the plastics and coating sectors, and metal smelting [4, 5]. In the soil of mainland China, concentrations of cadmium vary widely, from 0.003 mg/kg to 9.57 mg/kg [6]. Within fresh water, cadmium residues are normally detected at levels of 10 to 500 ng/L, but this contrasts starkly with the highest level observed in industrialized regions, in excess of 17 mg/L [7]. For occupationally exposed human males, the average concentration of cadmium is reportedly 1.18 μg/dl, while the highest concentration has been noted at 24 μg/dl [8]. Due to its long half-life and low excretion rate, accumulation of cadmium in various tissues and organs may link to hepatorenal toxicity, carcinogenicity, endocrine and reproductive toxicity [9]. Cadmium is also considered as an endocrine disrupting chemical that can directly or indirectly interfere with the generation, transport and excretion of endogenous hormones or the process of sperm production, thereby impairing male reproductive health [10].

Leydig cells are the primary producer of androgens in testes and are involved in maturation of spermatogenic cells and maintenance of reproductive health [11]. Testosterone is secreted by Leydig cells via the coordination of mitochondria and smooth endoplasmic reticulum. It is highly significant in both metabolic regulation and the maintenance of sexual function [11]. It has been reported that plasma testosterone levels and testosterone synthase activity decreased after pubertal cadmium exposure, leading to defects in development of adolescent testes and reproductive dysfunction in adult mice [12]. Animal studies demonstrated that cadmium exposure resulted in testicular toxicity including abscission of epithelial cells, apoptosis of testicular germ cells and lower level of testosterone, eventually leading to spermatogenesis defects and declined sperm quality [13, 14]. Nonetheless, the implications of cadmium-related damage to Leydig cells have received scant attention [15, 16], and the underlying mechanism of cytotoxicity and apoptosis remains to be elucidated.

Reportedly, from spermatogenesis to fertilization, the regulation of the physiological aspects of reproductive function is strongly influenced by mitochondria [17]. The latter are important dynamic organelles and undergo regulated cycles of fusion and fission. In mammalian cells, optic atrophy protein 1 (OPA1) and Mitofusin 1 (MFN1) are among the principal factors that influence mitochondrial fusion. By contrast, mitochondrial fission 1 protein (FIS1) and dynamin-related protein 1 (DRP1) are the key mediators of mitochondrial fission. Fusion helps to establish interconnected mitochondrial networks while fission generates fragmented mitochondria [18]. Mitochondrial dynamics is associated with various cellular processes, including mitochondrial quality control, metabolic adaptation, regulation of mitophagy and apoptosis [18]. Cadmium has been found to inhibit testosterone production and viability of rat Leydig cells, probably via interfering with mitochondrial function [15]. Thus, it is important to understand the effect of cadmium on mitochondrial dynamics, function and the cell fate of Leydig cells. In the present study, a mice model was used to evaluate the reproductive toxicity of cadmium. Moreover, TM3 cells were exposed to a range of cadmium concentrations to investigate the impact of cadmium on mitochondrial dynamics and to illuminate the mechanism of cadmium-induced apoptosis of Leydig cells.

## Materials and methods

### Ethics statement

All animal experiments were carried out in line with the regulations approved by the Animal Protection and Experimental Ethics Committee of Wuhan University of Science and Technology (approval No. 20180923, Wuhan, China). Animals were treated humanely in accordance with institutional animal welfare guidelines.

### Chemicals and reagents

Cadmium chloride (CAS:10108-64-2) with 99% analytical standard was obtained from Sigma-Aldrich (St. Louis, USA). Carbonyl cyanide 3-chlorophenylhydrazone (CCCP) and Mitochondrial division inhibitor 1 (Mdivi-1) were purchased from Beyotime Biotechnology Co, Ltd. (Beijing, China). The main antibodies used for Western blot analysis are listed as follows: Cytochrome c (CytC, #11940), Caspase-9 (#9508), Cleaved Caspase-3 (#9664), DRP1 (#8570), LC3A/B (#12741), TOM20 (#42406) and HSP60 (#12165) were purchased from Cell Signaling Technology (USA). Bax (50599-2-lg), BCL2 (26593-1-AP), PINK (23274-1-AP), Parkin (14060-1-AP), MFN1 (13798-1-AP), OPA1 (66583-1-lg), FIS1(10956-1-AP), SQSTM1/p62 (18420-1-AP), β-actin (66009-1-lg) and VDAC (10866-1-AP) were purchased from Proteintech (Wuhan, China). 3β-HSD (sc-515120) was purchased from Santa Cruz biotechnology. GAPDH (#AC033) was purchased from ABclonal (USA). CytC (#GB1108, Servicebio, China) and Parkin (14060-1-AP, Proteintech, China) were used for immunofluorescence analysis. The secondary antibody FITC (SA00003-2) and Cy3 (SA00009-1) was from Proteintech (Wuhan, China).

### Animals and experimental design

Male C57BL/6 mice aged 3 weeks were purchased from the animal center of Hubei Provincial Center for Disease and Prevention (China) and housed with ad libitum access to food and water during the experiment. The animal room was maintained at 22–25 °C with a 12 h light/dark cycle. Following preliminary experiments and published data [19, 20], twenty mice were randomly divided into four groups (*n* = 5 each) after 7 days of adaptive feeding. A free online randomization tool (https://www.graphpad.com/quickcalcs/randomize1.cfm) was used to allocate animals to the treatments. On the basis of cadmium levels detected in smokers and in individuals living in cadmium-contaminated areas, and following previous animal studies [21–24], mice in the high-dose group were injected intraperitoneally with 2.0 mg/kg BW of CdCl_2_. The selection of low-dose (0.5 mg/kg BW) and medium-dose (1.0 mg/kg BW) was determined in proportion to the high-dose group. Mice in the control group received intraperitoneal injection of normal saline. CdCl_2_ was dissolved in 0.9% NaCl and mice were injected intraperitoneally for four consecutive weeks. Mice were anesthetized and sacrificed at the end of the study. Blood was collected from the orbital vein and the serum was stored at −80 °C. The left side of the testis and epididymis were fixed with animal testicular tissue fixative (Servicebio, China). The other side of the testis was stored at −80 °C after snap-freezing in liquid nitrogen. After being collected from cauda epididymides in warm PBS, sperm were fixed with 2% formaldehyde for 10 min at room temperature. The total number of sperm was counted using a hemocytometer under an optical microscope at x200 magnification.

### H&E staining, immunohistochemistry and TUNEL analysis

After being dewaxed and rehydrated with xylene and gradient ethanol, 5 μm sections of paraffin embedded testis tissue were stained with hematoxylin and eosin (H&E) for morphological analysis. Immunohistochemistry was performed according to the methods previously described [12]. Leydig cells were identified by staining for 3β-hydroxysteroid dehydrogenase (3β-HSD) and the number of 3β-HSD-positive cells was counted in 25 fields randomly selected on slides in each group of mice. TUNEL staining was performed using one step TUNEL apoptosis assay kit (Meilunbio, China). To positively identify the apoptotic Leydig cells, the tissue sections were blocked with 10% goat serum and stained with 3β-HSD (1:50) for an additional 2 h at 37 °C after TUNEL staining. The sections were subsequently incubated with a goat anti-rabbit IgG Alexa fluor 647 conjugated antibody (1:100) for 1 h at 37 °C and observed using a laser confocal microscope.

### Isolation of mouse primary Leydig cells

Leydig cells were isolated from the testes of eight-week-old male C57BL/6 mice according to the previous methods [25]. The Leydig cells were cultured with DMEM/F-12 containing 10% FBS in a 5% CO_2_ incubator at 37 °C. Histochemical staining of 3β-HSD was used to determine the purity of the Leydig cells, which was found to be in excess of 90%. A one-step TUNEL apoptosis assay kit (Meilunbio, China) was employed to analyze the apoptosis of primary Leydig cells.

### Cell culture and treatment

Mouse Leydig cell line TM3 was purchased from Cell Bank (Shanghai Institute of Biological Sciences, China) and tested for mycoplasma contamination. The TM3 cells were maintained in DMEM/F-12 medium (Gibco, USA) with 5% horse serum (HAKATA, China) and 2.5% Fetal bovine serum (FBS) (Gibco, USA) at 37 °C and 5% CO_2_ in an incubator for routine culture. CdCl_2_ was dissolved in deionized water to produce a stock solution which was then used in cell culture medium.

### Cell counting kit 8 (CCK-8) assay

TM3 cells (8 × 10^3^ cells/well) were seeded in a 96-well culture plate and incubated for 24 h. After treatment with different concentrations of 0, 2.5, 5, 10, 20, 30 and 40 μM CdCl_2_ for 24 h, the cell viability was determined using Cell Counting Kit-8 (Meilunbio, China) according to the manufacturer’s instructions. The absorbance of each well at a wavelength of 450 nm was measured by a microplate reader (Bio-Tek, USA). Cell survival rate (%) = [(Cd experimental group - blank group)/(control group - blank group)]×100%.

### Testosterone concentration assay

Serum was collected from cadmium-exposed mice. The concentration of testosterone in the serum was measured according to the instructions of the enzyme-linked immunosorbent assay (ELISA) (Elabscence, China).

### Detection of apoptotic cells

Annexin V-FITC/PI Apoptosis Detection Kit (Beyotime, China) and Hoechst 33342 staining were used to determine apoptosis. The cells (1 × 10^5^) were resuspended in a 195 μl binding buffer containing 5 μl Annexin V-FITC and 10 μl PI, and incubated at room temperature for 15 min in the dark. The apoptosis was immediately measured by flow cytometry (BD Biosciences, USA) and analyzed by FlowJo software. In addition, detection of apoptosis was performed on adherent cells by incubation with Hoechst33342 for 10 min at 37 °C. The cells with condensed nuclei were counted from four different fields of each treatment and expressed as a percentage of total cells counted.

### Measurement of intracellular and mitochondrial reactive oxygen species (ROS)

2,7-Dichlorofuorescin Diacetate (DCFH-DA) (Nanjing Jiancheng Bioengineering Institute, China) and MitoSOX Red (Thermo Fisher Scientific, USA) were used to measure intracellular ROS and mitochondrial superoxide production, respectively. TM3 cells exposed to cadmium for 24 h were counted and incubated in 10 μM DCFH-DA and 2.5 μM MitoSOX at 37 °C for 30 min in the dark. The cells were analyzed by a multi-function microplate reader (Perkinelmer, USA).

### Measurement of mitochondrial membrane potential and ATP levels

JC-1 mitochondrial membrane potential measurement kits (Beyotime, China) were used to measure mitochondrial membrane potential (ΔΨm). Briefly, TM3 cells (2 × 10^5^) were resuspended in 0.5 mL JC-1 working solution and incubated at 37 °C for 20 min. The cells were washed with buffer twice and then analyzed by flow cytometry (BD Biosciences, USA). A luciferase-based enhanced ATP assay kit (Beyotime, China) was used to determine ATP levels. Briefly, the cells were washed with cold PBS in 6-well plates and lysed immediately in 200 μL lysis buffer on ice. The lysate was centrifuged at 12,000 *g* for 5 min. 20 μL of the supernatant was added into a 96-well plate containing 100 μL ATP detection working solution. The luminescence was detected by a multi-function microplate reader (Perkinelmer, USA). The protein concentration of each group and an ATP standard curve were used to calibrate the ATP levels in the cells. The cellular ATP levels were presented as the percentage of the level observed in the control group.

### Measurement of mitochondrial DNA (mtDNA) copy number and mitochondrial calcium

According to methods previously described [26], mtDNA copy number was analyzed by the real-time quantitative PCR with SYBR Green I. The mtDNA primers for *Cox1* (mitochondrially encoded cytochrome c oxidase I) were 5′-CCACTTCGCCATCATATTCGTAGG-3′ and 5′-TCTGAGTAGCGTCGTGGTATTCC-3′. The primers for *Gapdh* were 5′-AACCTGCCAAGTATGATGA-3′ and 5′-GAGTCCTTCCACGATACC-3′. The mtDNA copy number was determined as the ratio of *Cox1* to *Gapdh*. The concentration of mitochondrial Ca^2+^ in TM3 cells was determined using a fluorescent Ca^2+^ indicator Rhod-2 AM (Maokang Biotechnology, China). The cells were loaded with HBSS buffer (without Ca^2+^ and Mg^2+^) containing 4 µM Rhod-2 AM and incubated at 37 °C for 30 min in the dark. The cells were washed and incubated with HBSS buffer for 30 min. Cellular images were captured using a confocal microscope (Olympus, Japan).

### Mitochondrial staining and mitochondrial morphology analysis

TM3 cells were seeded on a laser confocal culture dish at the density of 1 × 10^5^ cells/mL and treated with cadmium for 24 h. The cells were stained with 200 nM Mito-Tracker Red CMXRos (Beyotime, China) for 30 min in the dark. The images were optimized for morphometric analysis using methods as previously described [27]. Briefly, mitochondria were isolated from background by applying a threshold to the image using Otsu’s method and were subjected to particle analysis. The aspect ratio and the form factor, which represent mitochondrial length and branching respectively, were calculated in individual cells. Between 20 and 30 cells (>500 mitochondria) were analyzed per treatment. Tubular or filamentous mitochondria were regarded as normal whereas dotted mitochondria were deemed to be damaged. The cells showing indicated mitochondrial morphology was counted and presented as the percentage of the total number of cells counted (at least 100 cells per experiment). The cells were evaluated by two blind observers.

### Immunofluorescence and colocalization analysis

After TM3 cells had been seeded on a confocal culture dish at 1 × 10^5^ cells/mL and treated with cadmium for 24 h, mitochondrial morphology was detected according to the Mito-Tracker Red CMXRos instructions. Subsequently, the cells were fixed with 4% paraformaldehyde and incubated with primary antibodies at 4 °C overnight, and then stained with FITC-labeled secondary antibodies for 1 h at room temperature. The samples were analyzed with a confocal microscope (Olympus, Japan). Colocalization was analyzed in ImageJ and presented as Pearson’s correlation coefficient [28].

### Subcellular fractionation and Western blotting analysis

The cytosolic and mitochondrial fractions were extracted from cells using a cell-mitochondria isolation kit (Beyotime, China). Protein concentration was quantified using a BCA protein assay kit (Meilunbio, China). Equal amounts (~35 μg/lane) of protein from each subcellular fraction were separated by SDS-PAGE and then electrophoretically transferred onto PVDF membranes (Millipore, USA). After blocking with 5% nonfat milk for 2 h, the membrane was probed with anti-CytC (1:1000), anti-Caspase-9 (1:1000), anti-Caspase-3 (1:1000), anti-Bax (1:5000), anti-BCL-2 (1:1000), anti-PINK (1:600), anti-Parkin (1:200), anti-LC3 (1:1000), anti-p62 (1:1000), anti-MFN1 (1:1000), anti-OPA1 (1:500), anti-DRP1 (1:500), anti-FIS1 (1:600), anti-GAPDH (1:80000) and anti-β-actin (1:20000) or anti-VDAC (1:1000) overnight at 4 °C. The membrane was washed with TBST buffer, followed by incubating with a goat anti-rabbit (or anti-mouse) secondary antibody conjugated with horseradish peroxidase (HRP) for 2 h at room temperature. Blots were revealed with enhanced chemiluminescent reagents (Meilunbio, China).

### Statistical analysis

Data were expressed as means ± SD and analyzed using GraphPad Prism 8.0 software. Differences in mean values between multiple groups were analyzed by one-way analysis of variance (ANOVA) with Bonferroni’s multiple comparisons test. A value of *P* < 0.05 was considered as statistically significant.

## Results

### Cadmium induced testicular toxicity in mice

**Fig. 1:**
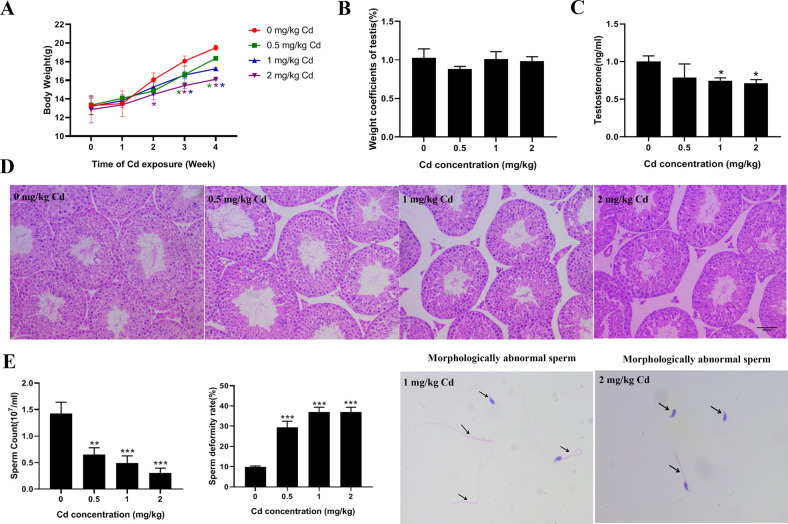
The effect of cadmium exposure on testicular toxicity. **A** The body weight of mice was recorded every day during the exposure period and weekly body weight was shown (*n* = 5 in each group). **B** Testis/body weight index of four groups. **C** Measurement of serum testosterone levels in four groups of mice. **D** Representative histology of the testis from each group, scale bar: 20 μm. **E** The number of sperm from cauda epididymides and deformity rate were examined, and abnormal sperm morphology was shown. Arrows indicate morphologically abnormal sperm. (*P < 0.05, **P < 0.01, ***P < 0.0001 vs. control).

During the period of exposure, the body weight of mice decreased from the second week after treatment with 2 mg/kg cadmium whereas it declined from the third week after treatment with 0.5 and 1 mg/kg cadmium (Fig. 1A). However, there were no significant differences in testis/body weight among the four groups at the end of exposure (Fig. 1B). The serum testosterone levels showed a downward trend and significantly decreased in 1 mg/kg and 2 mg/kg cadmium groups (Fig. 1C). The testicular histological morphology was examined to evaluate testicular toxicity induced by cadmium. H&E staining showed that the structure of seminiferous tubules and organization of germ cells were normal in the control group. However, the interstitial space between seminiferous tubules was enlarged in the cadmium groups. Additionally noticeable were abscission of spermatogenic cells, disorganization of seminiferous tubules with vacuoles, and disordered arrangement of spermatid cells (Fig. 1D). Furthermore, cadmium exposure resulted in a decrease in sperm counts and increased rate of sperm deformity. Sperm with bent or short tails, sperm without tails or heads, and other morphological abnormalities were observed (Fig. 1E).

### Cadmium reduced the number of Leydig cells and induced cell apoptosis

**Fig. 2:**
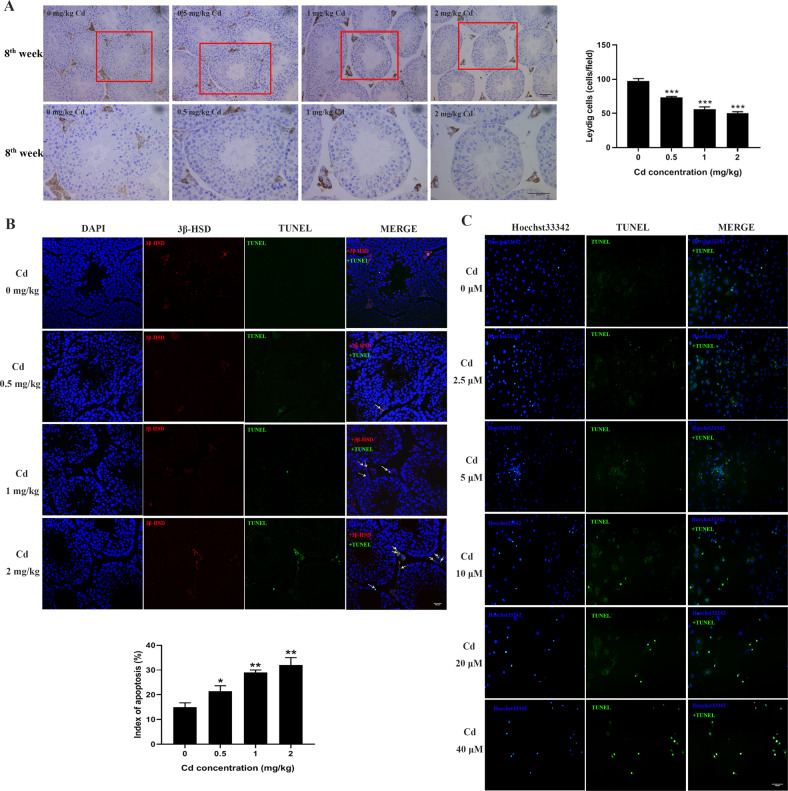
Cadmium exposure decreased the number of Leydig cells and induced apoptosis in testes. **A** Representative images of testis immunohistochemistry with anti-3β-HSD. The Leydig cells were stained in brown, scale bar: 20 μm. The number of 3β-HSD-positive cells was counted in 25 fields randomly selected on slides in each group of mice. **B** Testis immunohistochemistry combined TUNEL staining to analyze apoptosis of Leydig cells in vivo. White arrows indicate apoptotic Leydig cells. Broken white arrows indicate apoptotic spermatogenic cells. scale bar: 50 μm (**C**) Primary Leydig cells were exposed to different concentrations of cadmium and cell apoptosis was analyzed by Hoechst33342 and TUNEL staining, scale bar: 100 μm.

To explore the effect of cadmium exposure on the viability of Leydig cells, immunohistochemistry was performed with a Leydig cell-specific marker 3β-HSD. As shown in Fig. 2A, the number of Leydig cells in the cadmium-exposed mice was significantly lower than that in the control group. We performed TUNEL in conjunction with immunohistochemistry to determine whether cadmium treatment induces apoptosis of Leydig cells in vivo. A number of TUNEL-positive Leydig cells were observed in the testes of the cadmium-exposed mice (Fig. 2B). Moreover, Following treatment with different concentrations of cadmium in vitro, condensed chromatin and apoptotic bodies were displayed in isolated primary Leydig cells via Hoechst and TUNEL staining (Fig. 2C). These results suggest a correlation between cadmium exposure and apoptosis of Leydig cells in mice.

### Cadmium inhibited proliferation and induced apoptosis of TM3 cells

**Fig. 3:**
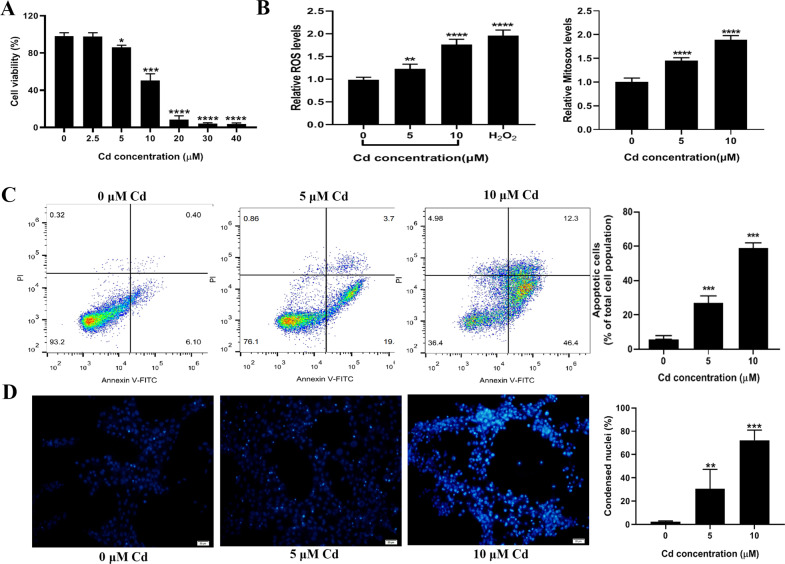
Cadmium exposure decreased cell viability and induced ROS and apoptosis of TM3 cells. **A** TM3 cells were exposed to different concentrations of cadmium for 24 h. The cell viability was measured by CCK-8 assays. **B** Cellular ROS and Mitochondrial ROS production. **C** Apoptotic cells were detected by PI and Annexin V staining and represented as a percentage ratio. **D** Representative images of nuclear staining with Hoechst33342 in TM3 cells. Apoptotic cells were quantitated by nuclear condensation. Data represent the mean±SD for at least three independent experiments (**P < 0.01, ***P < 0.001, ****P < 0.0001 vs. control).

The mouse Leydig cell line TM3 was selected as an in vitro model system to study the mechanism of cadmium-induced apoptosis of Leydig cells. The cells were cultivated with different concentrations of cadmium (0, 2.5, 5, 10, 20, 30 and 40 μM) for 24 h and the cell viability was measured by CCK-8 assays. The viability of TM3 cells was reduced in a dose-dependent manner and the IC_50_ of cadmium was calculated to be 10.01 μM (1.84 μg/mL), 95% CI [9.58, 10.47] (Fig. 3A). The mean cadmium level of 0.56 μg/g has been reported in human testes from 41 autopsy cases [29]. Moreover, it was reported that cadmium was accumulated to levels of around 14 μg/g in mouse testes after five weeks of exposure to 0.8 mg/kg CdCl_2_ [19]. Based on these results, the cadmium concentrations of 0, 5 and 10 μM were used in the following experiments. Next, we measured ROS levels and apoptosis in the TM3 cells. As shown in Fig. 3B, levels of cellular ROS (measured using DCFH-DA) and mitochondrial superoxide (measured by MitoSOX) increased in the cadmium-treated cells. Compared to the control group, cell apoptosis increased significantly at cadmium concentrations of 5 and 10 μM (Fig. 3C). Likewise, among the cells exposed to cadmium, a higher percentage of cells with nuclear condensation was observed as by Hoechst33342 staining (Fig. 3D).

### Cadmium induced mitochondrial fragmentation and dysfunction in TM3 cells

**Fig. 4:**
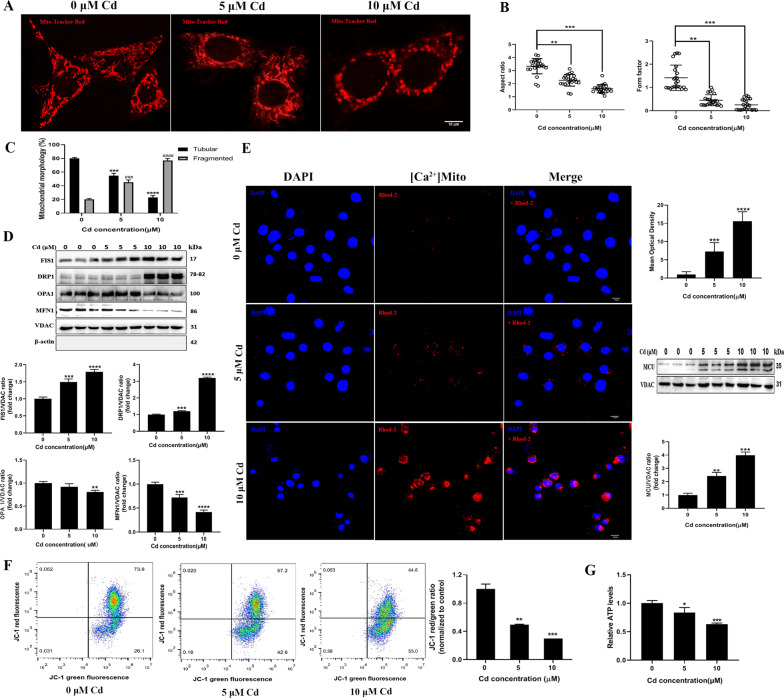
Cadmium induced mitochondrial fragmentation and dysfunction in TM3 cells. **A**, **B** Mitochondria were stained with Mito-Tracker Red CMXRos after the cells were treated with cadmium. The mitochondrial morphology factors (aspect ratio and form factor) were evaluated. **C** The percentage of cells containing tubular or fragmented mitochondria was calculated. **D** The protein levels of FIS1, DRP1, OPA1 and MFN1 were measured by Western blot after treatment with cadmium. **E** The effects of cadmium on mitochondrial Ca^2+^ levels and expression of mitochondrial calcium uniporter (MCU) were analyzed by immunofluorescence and Western blot, respectively. **F** The effect of cadmium on mitochondrial ΔΨm was assayed by JC-1 staining. **G** Luciferase-based assays were used to determine cellular ATP production. The data represent the mean ± SD of three independent experiments (*P < 0.05, **P < 0.01, ***P < 0.001, ^###^*P* < 0.001, ****>*P* < 0.0001, ^####^*P* < 0.0001 vs. control).

To clarify the impact of cadmium on mitochondrial morphology in TM3 cells, mitochondria were stained with MitoTracker Red dye. The morphology of mitochondria displayed a filamentous or tubular pattern, forming interconnected networks in the control group. By contrast, mitochondria were shortened and less connected in the cadmium-treated cells, which indicates mitochondrial fragmentation (Fig. 4A). Quantitative analyses of mitochondrial length (aspect ratio) and branching (form factor) confirmed the mitochondrial fragmentation (Fig. 4B). As concentrations of cadmium increased, the percentage of cells with fragmented mitochondria rose while that of cells with tubular mitochondria decreased (Fig. 4C). The results suggest dysregulation of mitochondrial fission and fusion events when the cells were exposed to cadmium. Thus, we extracted mitochondria from the cells and measured the expression of the main modulators of mitochondrial division machinery. Cadmium notably increased the expression levels of DRP1 and FIS1, two proteins that control mitochondrial fission. By contrast, levels of OPA1 and MFN1, which regulate mitochondrial fusion, were reduced by cadmium (Fig. 4D). These results indicate that cadmium may shift mitochondrial dynamics towards fission, leading to an increased mitochondrial fragmentation. The involvement of calcium homeostasis in apoptosis and the regulation of mitochondrial dynamics has been suggested by earlier research, we thus explored the influence of cadmium on the levels mitochondrial Ca^2+^ and the expression of mitochondrial calcium uniporter (MCU). The latter is located within mitochondria and mediates mitochondrial Ca^2+^ uptake. As shown in Fig. 4E, cadmium elevated mitochondrial Ca^2+^ level and significantly increased the protein level of MCU in the cells.

In addition, analysis of mitochondrial membrane potential (ΔΨm) and ATP production in the TM3 cells was undertaken to explore the effect of cadmium on mitochondrial function. JC-1 staining showed that cadmium sufficiently reduced ΔΨm (Fig. 4F). The ATP level was reduced by 17% and 37% when the cells were exposed to 5 μM and 10 μM cadmium, respectively (Fig. 4G). The data indicate that both mitochondrial depolarization and dysfunction were induced by cadmium in TM3 cells.

### Cadmium induced release of cytochrome c and caspase activation in TM3 cells

**Fig. 5:**
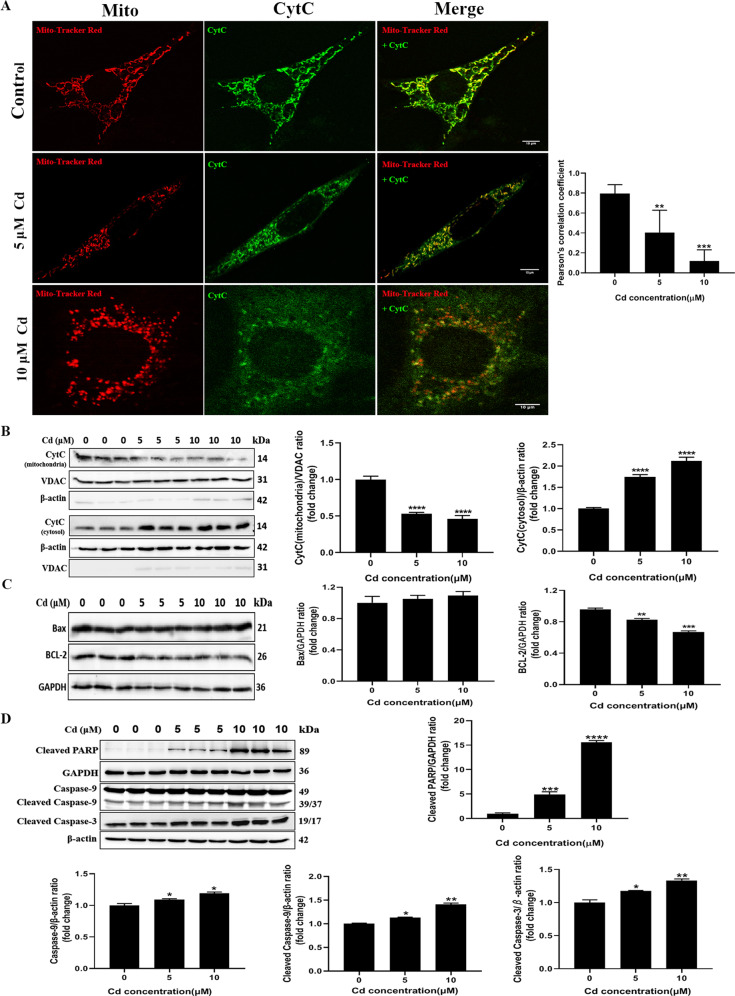
Cadmium induced the release of cytochrome c from the mitochondria into the cytosol of TM3 cells and promoted apoptosis. **A**, **B** The cellular distribution of cytochrome c was analyzed by immunofluorescence and Western blotting after the cells were treated with cadmium for 24 h. The cells were stained with mitochondrial probes (Mito-Tracker Red CMXRos), incubated with anti-CytC (Green) and then observed by laser confocal microscopy (100x). Pearson’s correlation coefficient was used to assess the colocalization between mitochondria and cytochrome c. VDAC and β-actin were used as internal references for mitochondrial and cytosolic fractions, respectively. **C**, **D** The levels of apoptosis-related proteins Bax, Bcl-2, cleaved PARP, Caspase-9, cleaved Caspase-9 and cleaved Caspase-3 were measured by Western blot. The data represent the mean ± SD of three independent experiments (**P < 0.01, ***P < 0.001, ****P < 0.0001 vs. control).

Mitochondrial fission is know to be linked with the release of cytochrome c, which regulates cell apoptosis. The influence of cadmium on the distribution of cytochrome c in TM3 cells was analyzed by immunofluorescence. Cytochrome c was co-localized with filamentous mitochondria in the control, suggesting it is present in the mitochondria. By contrast, cadmium treatment resulted in mitochondrial fragmentation and cytochrome c became visible in the cytosol. The Pearson’s correlation coefficient for colocalization between mitochondria and cytochrome c declined significantly in cadmium-treated cells compared to the control group (Fig. 5A). Next, we separated the mitochondria from the cytoplasm of TM3 cells and determined the protein level of cytochrome c in these cellular fractions. Consistently, the level of cytochrome c in the mitochondrial fraction decreased significantly while it increased greatly in the cytosolic fraction with increasing concentrations of cadmium (Fig. 5B). Furthermore, the expression of anti-apoptotic Bcl-2 and pro-apoptotic Bax, which are implicated in regulation of cytochrome c release, was measured by Western blot. Bcl-2 levels declined in response to cadmium exposure while Bax remained at similar levels in all groups (Fig. 5C). Since the release of cytochrome c from mitochondria to cytosol can trigger the caspase cascade and initiate apoptosis, the expression levels of apoptosis-related proteins were determined. As shown in Fig. 5D, cadmium led to an increase in the level of cleaved PARP, Caspase-9, cleaved Caspase-9 and cleaved Caspase-3. These results imply that cytochrome c, released from the damaged mitochondria of TM3 cells, promoted the cadmium-induced apoptosis via activation of the caspase cascade.

### Inhibition of mitochondrial fission obstructed the release of cytochrome c and apoptosis of TM3 cells

**Fig. 6:**
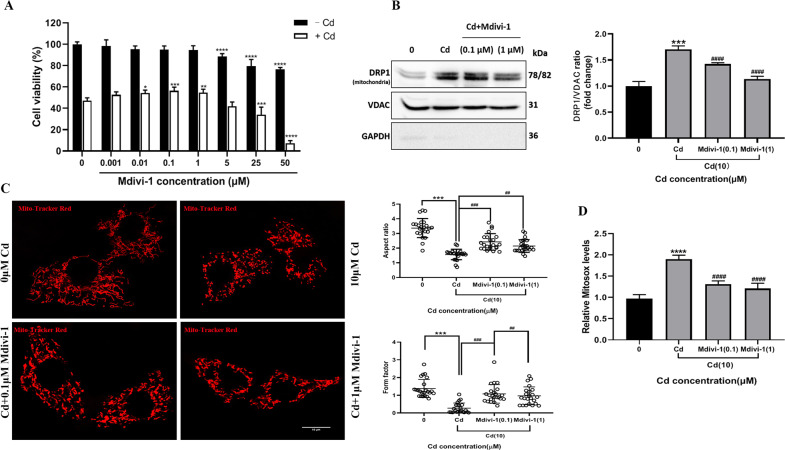
The DRP1 inhibitor Mdivi-1 protected mitochondrial damage caused by cadmium. **A** TM3 cells were pretreated with a range of Mdivi-1 concentrations, followed by exposure to 10 μM Cd for 24 h and the cell viability was measured by CCK-8 assays. **B** The effect of Mdivi-1 on the mitochondrial location of DRP1 was determined by Western blot. VDAC and GAPDH were used as internal references for mitochondrial and cytosolic fractions, respectively. **C** The effect of Mdivi-1 on cadmium-induced mitochondrial deformation was analyzed by MitoTracker Red staining, and the aspect ratio and form factor were assessed. **D** Measurement of mitochondrial ROS levels. Data represent the mean±SD for at least three independent experiments (**P < 0.01, ***P < 0.001, ****P < 0.0001 vs. control; ^####^*P* < 0.0001 vs. Cd).

To examine whether cadmium-induced mitochondrial deformation leads to cell apoptosis, TM3 cells were treated with Mdivi-1 which inhibits mitochondrial fission by deactivation of DRP1 [30]. Based on the results of CCK-8 assays, we chose 0.1 and 1 μM Mdivi-1 for the following experiments because the cell viability was not significantly reduced at these concentrations in the absence of cadmium (Fig. 6A). Addition of Mdivi-1 caused a decrease in cadmium-induced mitochondrial DRP1 levels (Fig. 6B). In comparison with the cells treated exclusively with cadmium, fragmented mitochondria partially reverted to normal morphology, and both aspect ratio and form factor increased (Fig. 6C). Mdivi-1 also decreased mitochondrial superoxide production of cadmium-treated cells (Fig. 6D).

**Fig. 7:**
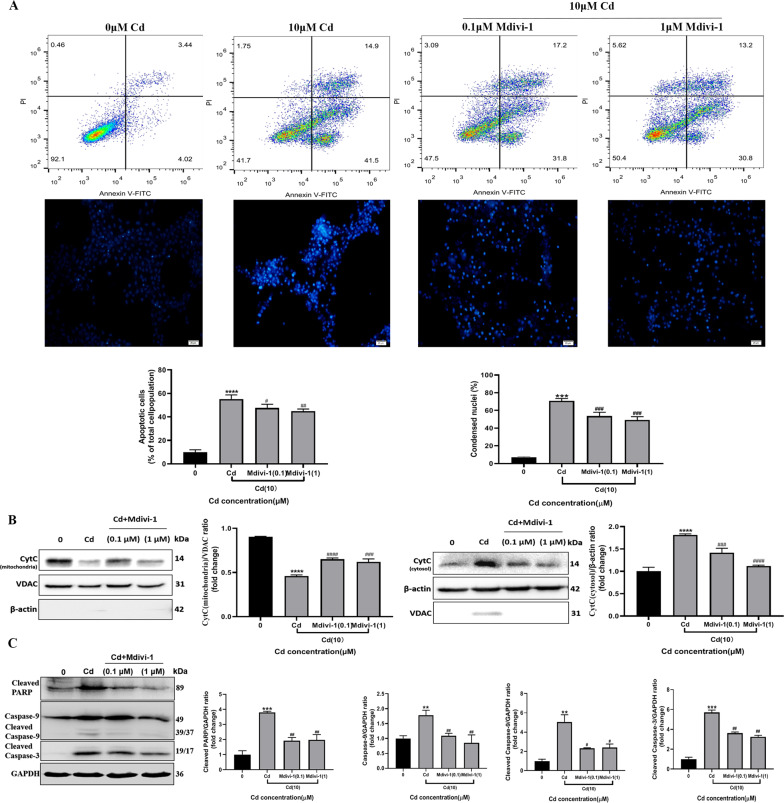
Mdivi-1 attenuated cadmium-induced apoptosis of TM3 cells. **A** The effect of Mdivi-1 on cadmium-induced apoptosis was evaluated by PI/Annexin V staining and Hoechst33342 staining, respectively. **B** Cellular distribution of cytochrome c was analyzed by Western blot. VDAC and β-actin were used as internal references for mitochondrial and cytosolic fractions, respectively. **C** Mdivi-1 inhibited cadmium-induced expression of cleaved PARP, Caspase-9, cleaved Caspase-9 and cleaved Caspase-3. Data represent the mean±SD for three independent experiments (**P < 0.01, ***P < 0.001, ****P < 0.0001 vs. control; ^#^*P* < 0.05, ^##^*P* < 0.01, ^###^*P* < 0.001, ^####^*P* < 0.0001 vs^.^ Cd).

In addition, we investigated whether Mdivi-1 could attenuate the cadmium-induced apoptosis of TM3 cells. Mdivi-1 inhibited cadmium-induced apoptosis compared to cadmium exposure alone (Fig. 7A). It increased levels of cytochrome c in the mitochondrial fraction but decreased them in the cytosolic fraction of cadmium-treated cells (Fig. 7B). Consistently, Mdivi-1 reduced cadmium-induced expression of cleaved PARP, Caspase-9, cleaved Caspase-9 and cleaved Caspase-3 (Fig. 7C). These data suggest that inhibition of mitochondrial fission by Mdivi-1 could prevent mitochondrial translocation of DRP1 and the release of cytochrome c from mitochondria, which in turn decreases cadmium-induced apoptosis.

### Cadmium-induced mitochondrial fragmentation was linked to inhibition of mitophagy

**Fig. 8:**
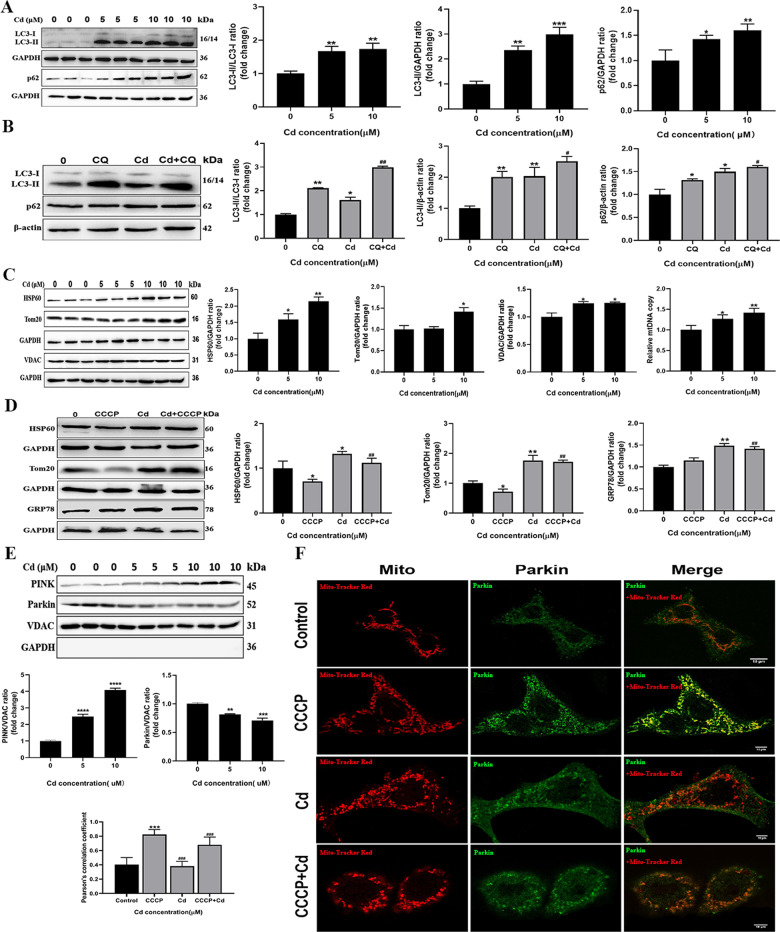
Cadmium-induced mitochondrial fragmentation was associated with inhibition of mitophagy. **A** Expression levels of mitophagy markers LC3 and p62 were examined by Western blot after TM3 cells were treated with cadmium. **B** The cells were treated with or without chloroquine (CQ, 10 μM) in the presence or absence of cadmium for 24 h. The expression of LC3 and p62 was measured. **C** The effect of cadmium on mitochondrial mass was analyzed by immunoblotting for mitochondrial proteins (HSP60, TOM20 and VDAC) and measuring mitochondrial DNA (mtDNA) copy number. **D** The cells were exposed to 10 μM cadmium for 24 h with or without pretreatment with 10 μM CCCP for 2 h. The levels of mitochondrial proteins (HSP60 and Tom20) and an ER protein GRP78 were measured. **E** Mitochondria were isolated from the cadmium-treated cells and the expression of PINK and Parkin were measured by Western blot. VDAC and GAPDH were used as internal references for the mitochondria and cytosol, respectively (**F**) The cells were treated with cadmium and CCCP as above and then stained with mitochondrial probes (Mito-Tracker Red CMXRos) and anti-Parkin antibodies (Green). The colocalization between mitochondria and Parkin was analyzed by immunofluorescence. Data represent the mean±SD for at least three independent experiments (*P < 0.05, **P < 0.01, ***P < 0.001, ****P < 0.0001 vs. control; ^##^*P* < 0.01, ^###^*P* < 0.001, vs. CQ or CCCP).

It is known that mitophagy is involved in elimination of damaged mitochondria to maintain cellular homeostasis [31]. In cadmium-treated TM3 cells, mitochondrial dysfunction and excessive mitochondrial fragmentation were detected, suggesting mitophagy could be compromised. To study the effect of cadmium on mitophagy in the cells, the protein levels of autophagy-related markers, LC3 and p62, were firstly analyzed by Western blot. As shown in Fig. 8A, the levels of LC3-II and p62 elevated in the cadmium-treated cells compared to the control. Next, we measured levels of LC3-II and p62 in the presence of chloroquine (CQ), which inhibits lysosomal degradation by blocking fusion between atuophagosomes and lysosomes. The protein levels of LC3-II and p62 increased after treatment with cadmium or CQ respectively, compared to the control group. Administration of both cadmium and CQ further increased the levels of these proteins (Fig. 8B). This implies that cadmium treatment inhibits autophagic flux in the cells. Furthermore, the effect of cadmium on mitochondrial mass was assessed by immunoblotting for mitochondrial proteins HSP60, TOM20 and VDAC. Cadmium treatment caused a significant increase in the expression levels of these proteins. Consistent with this finding, mitochondrial DNA (mtDNA) copy number in the cadmium-treated cells was higher than that in the untreated cells. These data suggest that cadmium treatment results in the accumulation of mitochondrial structure and impairment of mitophagy in TM3 cells (Fig. 8C).

To confirm the effect of cadmium on mitophagy, CCCP was utilized to induce mitophagy in TM3 cells. The levels of mitochondrial protein HSP60 and Tom20 in the CCCP-treated cells were significantly lower than that in the control, indicating the removal of damaged mitochondria by mitophagy. However, CCCP-induced reduction in the levels of HSP60 and Tom20 were reversed by cadmium (Fig. 8D). These findings indicate that cadmium probably blocked autophagic flux via inhibition of autophagosome-lysosome fusion in the cells, thereby interrupting mitophagy. Moreover, the effect of cadmium on expression of GRP78, a marker of endoplasmic reticulum stress, was evaluated. The level of GRP78 slightly increased in CCCP-treated cells, whereas it was elevated in the presence of cadmium (Fig. 8D). It is likely that cadmium causes endoplasmic reticulum stress in the cells.

Next, we isolated mitochondria from TM3 cells and levels of PINK1 and Parkin, both of which are implicated in mitophagy, were examined. Under normal conditions, PINK1 is rapidly degraded by proteolysis and is maintained at low levels in healthy mitochondria. When mitochondria are damaged and lose ΔΨm, PINK1 is stabilized on the outer mitochondrial membrane and recruits Parkin to activate mitophagy for elimination of damaged mitochondria [31]. As shown in Fig. 8E, increased level of PINK1 and reduced level of Parkin were detected in the mitochondrial fraction of cadmium-treated cells. This implies the impairment of mitophagy in the cells. To confirm the role of cadmium in blocking of Parkin translocation to damaged mitochondria, the distribution of Parkin in TM3 cells was investigated by immunofluorescence. Compared to the control group, the extent of colocalization of mitochondria with Parkin substantially increased in the CCCP-treated cells, indicating recruitment of Parkin to the mitochondria. By contrast, cadmium promoted mitochondrial fragmentation and decreased colocalization of mitochondria with Parkin in the CCCP-treated cells (Fig. 8F). These data indicate that Parkin was not efficiently recruited to damaged mitochondria in the cadmium-treated cells, even though PINK1 was accumulated on the mitochondria with reduced ΔΨm. As a result, mitophagy was compromised.

## Discussion

Cadmium is an environmental contaminant derived from industrial activities. It has been reported that cadmium is capable of disrupting the testicular tissue structure and causing abnormal spermatogenesis with reduced level of testosterone [13]. As the primary producer of androgen, Leydig cells are vital to maintain normal development of spermatogenesis. However, current research rarely explores the underlying mechanism of cadmium-induced toxicity and apoptosis in these cells. This study demonstrated that cadmium exposure led to defects in the seminiferous epithelium, aberrant spermatozoa and apoptosis of Leydig cells in mice. The cadmium-induced apoptosis of mouse Leydig cells is probably caused by the induction of excessive mitochondrial fission and the inhibition of mitophagy.

Mitochondria continuously undergo regulated cycles of fusion and fission that are necessary for the maintenance of mitochondrial function and cellular homeostasis in respond to environmental stimuli [32]. Mitochondrial fission provides quality control by segregating damaged/dysfunctional components from the healthy mitochondrial network [32]. It has been reported that ROS are mainly generated in damaged mitochondria and separation of dysfunctional mitochondria for elimination could prevent further oxidative damage to the cells [33, 34]. Mitochondrial fusion is thought to rescue impaired mitochondria via quickly equilibrating mtDNA copies, matrix and membrane components, and is thus critical for maintenance of mitochondrial function [35, 36]. In this study, the expression of mitochondrial fusion factors OPA1 and MFN1 decreased apparently while the expression of DRP1 and FIS1 (mitochondrial fission mediators) increased significantly in the mitochondrial fraction of cadmium-treated cells. Consequently, the dynamic balance of fusion and fission events was disrupted by cadmium, causing mitochondrial fragmentation and mitochondrial dysfunction, as evinced by increased ROS levels and reduced ΔΨm and ATP production. These findings are consistent with earlier research that DRP1-dependent mitochondrial fission contributes to cadmium-induced hepatotoxicity [30, 37]. The mitochondrial damage caused by excessive mitochondrial fragmentation could partially explain the induced oxidative stress, opening of the mitochondrial permeability transition pore, inhibition of respiratory chain reaction and reduced ATP contents [37–39].

DRP1, recruited from cytosol to mitochondria through MFF and MiD49/51 (its adaptors on the outer membrane), is a key regulator of mitochondrial fission [18]. It has an antagonistic effect on the activity of anti-apoptotic protein Bcl-2 probably via competition for binding to FIS1, even though mitochondrial permeability transition and the release of cytochrome c are blocked by Bcl-2 [40]. Moreover, DRP1 is able to stimulate oligomerization of a pro-apoptotic protein Bax in the mitochondrial outer membrane. This, in turn, induces mitochondrial outer membrane permeability (MOMP) and the release of cytochrome c during apoptosis [41]. The increased expression of DRP1 and lower level of Bcl-2 are probably linked to induction of MOMP in cadmium-treated TM3 cells. Conversely, some studies have suggested that mitochondrial fission appears to be unnecessary for MOMP and cytochrome c release, and mitochondrial fusion-fission dynamics and apoptosis could be uncoupled [42, 43]. Regardless of conflicting findings, the present study showed that cadmium exposure led to accumulation of DRP1 in the mitochondria, release of cytochrome c from mitochondria to cytosol and cell apoptosis. Inhibition of mitochondrial fission by treatment with DRP1 inhibitor (Mdivi-1) attenuated cadmium-induced accumulation of mitochondrial superoxide and partially prevented cytochrome c release and apoptosis. Consistently, other models have demonstrated that downregulation of DRP1 by RNAi or overexpression of dominant-negative DRP1^K38A^ are effective in blocking mitochondrial fission and cell apoptosis [44, 45]. The present study did not explore the effect of FIS1 inhibition on cadmium-induced apoptosis of TM3 cells. Nonetheless, FIS1 knockdown reportedly compromises exogenous stimuli-induced mitochondrial fission and apoptosis [46, 47]. Knockdown of DRP1 expression inhibits stimuli-induced apoptosis to extent lower than down-regulation of FIS1 [47]. Our data suggest that the cadmium-induced mitochondrial fission contributes to mitochondrial dysfunction and activation of mitochondria-dependent apoptosis in TM3 cells.

Calcium, an intracellular signaling messenger, is stored mainly in the endoplasmic reticulum and mitochondria. Mitochondria-associated membranes facilitate the transfer of Ca^2+^ signals from the ER to mitochondria, and these signals are converted to specific stimuli, which in turn regulate diverse biochemical and cellular processes [48]. During stress, Ca^2+^ homeostatic imbalance results in the prolonged release of Ca^2+^ into mitochondria, which causes a decline of mitochondrial membrane potential and ultimately activates the intrinsic apoptosis [48]. Cadmium treatment has shown to induce imbalance of Ca^2+^ homeostasis and ER stress, thereby promoting hepatotoxicity [37, 49]. Mitochondrial Ca^2+^ overload could lead to abnormal mitochondrial dynamics and induction of apoptosis [50]. The results of our study demonstrated that the mitochondrial Ca^2+^ content and the expression of MCU in cadmium-treated TM3 cells were notably higher than those without treatment, indicating cadmium-induced alteration of Ca^2+^ homeostasis. Furthermore, dysregulation of calcium homeostasis is commonly associated with ER stress, along with Ca^2+^ depletion in the ER and the elevated expression of GRP78 [51]. The induced expression of GRP78 in the cadmium-treated cells suggests the occurrence of ER stress. Therefore, the specific mechanism underlying disturbed calcium homeostasis and ER stress-induced apoptosis remains to be further investigated in TM3 cells.

As an important process of mitochondrial quality control, autophagy/mitophagy maintains mitochondrial homeostasis and is implicated in the regulation of cell fate [31]. Autophagy was found to prevent against apoptosis of neuronal PC12 cells exposed to cadmium [52]. Conversely, cadmium-induced autophagy/mitophagy contributes to cytotoxicity rather than cellular survival in mouse brain neurons and kidney cells [53, 54]. Thus, the functional role of mitophagy in the cell fate is likely dependent on the different types of cells. In the present study, cadmium exposure resulted in blocking of Parkin recruitment to the mitochondria and inhibition of autophagic flux, which implies impaired mitophagy in TM3 cells. Consequently, the damaged mitochondria were not subject to Parkin-mediated autophagosome engulfment for degradation, thus generating excessive ROS and cell death. Further studies are required to clarify the mechanism by which cadmium inhibits the recruitment of Parkin to the mitochondria.

**Fig. 9:**
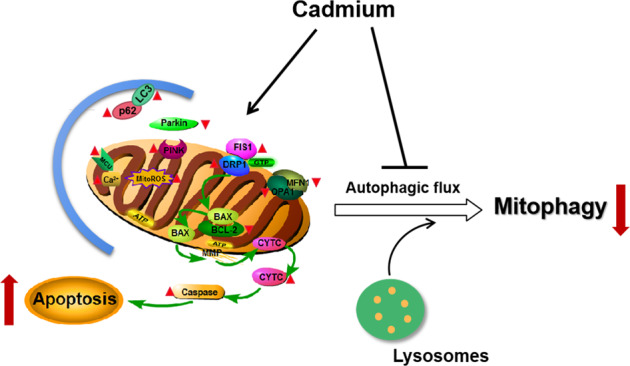
Schematic illustration of cadmium-induced toxicity in TM3 cells. Cadmium causes mitochondrial dysfunction and promotes excessive mitochondrial fission by controlling the expression of mitochondrial dynamics regulatory proteins (DRP1, FIS1, MFN1 and OPA1). Consequently, cytochrome c is released from the mitochondria to cytosol and the mitochondria-dependent intrinsic apoptosis of TM3 cells is induced. Moreover, cadmium probably disrupts calcium homeostasis and inhibits autophagic flux in the cells. The blocking of Parkin recruitment into damaged mitochondria by cadmium results in inhibition of mitophagy, production of excessive ROS and cell death.

In summary, the present findings indicate that the alternation of mitochondrial dynamics and inhibition of mitophagy contribute to cadmium-induced cytotoxicity of Leydig cells (Fig. 9). Cadmium treatment causes excessive mitochondrial fragmentation, followed by mitochondrial dysfunction and release of cytochrome c. This may induce mitochondria-dependent intrinsic apoptosis. Moreover, cadmium probably disrupts calcium homeostasis and inhibits autophagosome-lysosome fusion via unknown mechanisms. Further research is necessary to explore the underlying mechanisms through which mitophagy is implicated in the apoptosis of Leydig cells.

## Supplementary information

aj-checklist

## Data Availability

All data generated during the current study are included in this manuscript and are available from the corresponding author on reasonable request.
